# UNDERSTANDING OLDER ADULTS’ DIGITAL EXPERIENCES DURING AND AFTER THE PANDEMIC: A MIXED-METHODS STUDY

**DOI:** 10.1093/geroni/igae098.0116

**Published:** 2024-12-31

**Authors:** Zhan Yu, Xiayu Chen, Qingwen Xu, Chenxi Huang

**Affiliations:** East China Normal University, Shanghai, Shanghai, China (People’s Republic); University of Illinois Urbana-Champaign, Champaign, Illinois, United States; New York University, New York, New York, United States; East China Normal University, Shanghai, Shanghai, China (People’s Republic)

## Abstract

During the COVID-19 pandemic, China adopted the zero-case policy and made full use of digital technology, such as mandatory QR code using; consequently, older adults were forced into the experiences of digital technology. This study aims to explore the psychological and behavioral effects of policy actions on older Chinese, particularly to address the long-term effects on their technology use. We employed a sequential mixed-methods approach. Using data from the CHRLS, generalized estimating equations was performed and the study found a significant increase in internet use among older adults in 2020 compared to 2015 (OR = 12.04, p <.001). Then three focus group interviews were conducted with older adults in Shanghai. Content analysis reveals three themes: the feeling of being “compelled” to use digital technologies during the pandemic to meet daily basic needs and maintain social connections; a perception of “digital invasion” in the post-pandemic era; and an increased consciousness of the importance of self-efficacy for continued technology use. Further codes network analysis and cross-group comparisons show that both positive and negative experiences of digital use during the pandemic related to the increased use after the pandemic; prior community technology education programs and support received resulted in varied experiences and perceptions of digital use. This study offers insights into older adults’ adaptations to technology, which would help inform policies and practices for addressing the challenges of aging and technology use. It also highlights the need for tailored strategies that recognize the diverse needs of older adults in the digital age.

